# DeepCUBIT: Predicting Lymphovascular Invasion or Pathological Lymph Node Involvement of Clinical T1 Stage Non-Small Cell Lung Cancer on Chest CT Scan Using Deep Cubical Nodule Transfer Learning Algorithm

**DOI:** 10.3389/fonc.2021.661244

**Published:** 2021-07-05

**Authors:** Kyongmin Sarah Beck, Bomi Gil, Sae Jung Na, Ji Hyung Hong, Sang Hoon Chun, Ho Jung An, Jae Jun Kim, Soon Auck Hong, Bora Lee, Won Sang Shim, Sungsoo Park, Yoon Ho Ko

**Affiliations:** ^1^ Department of Radiology, College of Medicine, The Catholic University of Korea, Seoul, South Korea; ^2^ Division of Oncology, Department of Internal Medicine, College of Medicine, The Catholic University of Korea, Seoul, South Korea; ^3^ Department of Thoracic and Cardiovascular Surgery, Uijeongbu St. Mary’s Hospital, College of Medicine, The Catholic University of Korea, Seoul, South Korea; ^4^ Department of Pathology, College of Medicine, Chung-Ang University, Seoul, South Korea; ^5^ Deargen Inc., Daejeon, South Korea; ^6^ Cancer Research Institute, College of Medicine, The Catholic University of Korea, Seoul, South Korea

**Keywords:** deep learning, non-small cell lung cancer, prognosis, computed tomography, lobectomy

## Abstract

The prediction of lymphovascular invasion (LVI) or pathological nodal involvement of tumor cells is critical for successful treatment in early stage non-small cell lung cancer (NSCLC). We developed and validated a Deep Cubical Nodule Transfer Learning Algorithm (DeepCUBIT) using transfer learning and 3D Convolutional Neural Network (CNN) to predict LVI or pathological nodal involvement on chest CT images. A total of 695 preoperative CT images of resected NSCLC with tumor size of less than or equal to 3 cm from 2008 to 2015 were used to train and validate the DeepCUBIT model using five-fold cross-validation method. We also used tumor size and consolidation to tumor ratio (C/T ratio) to build a support vector machine (SVM) classifier. Two-hundred and fifty-four out of 695 samples (36.5%) had LVI or nodal involvement. An integrated model (3D CNN + Tumor size + C/T ratio) showed sensitivity of 31.8%, specificity of 89.8%, accuracy of 76.4%, and AUC of 0.759 on external validation cohort. Three single SVM models, using 3D CNN (DeepCUBIT), tumor size or C/T ratio, showed AUCs of 0.717, 0.630 and 0.683, respectively on external validation cohort. DeepCUBIT showed the best single model compared to the models using only C/T ratio or tumor size. In addition, the DeepCUBIT model could significantly identify the prognosis of resected NSCLC patients even in stage I. DeepCUBIT using transfer learning and 3D CNN can accurately predict LVI or nodal involvement in cT1 size NSCLC on CT images. Thus, it can provide a more accurate selection of candidates who will benefit from limited surgery without increasing the risk of recurrence.

## Introduction

Lung cancer is one of the most prevalent lethal diseases in the world. Over the past decade, the percentage of early-stage lung cancer has also increased; clinical stage IA disease has increased to account for 15% of non-small cell lung cancer (NSCLC) patients in developed countries (1). The standard treatment for stage I NSCLC is lobectomy with mediastinal lymph node dissection for the best chance of cure (2). However, due to an increase of screen-detected, indolent cancers appearing as subsolid nodules, there has been a shift of surgical treatment modality toward limited resection, such as sublobar resection. Yet, randomized trials are ongoing and the results for limited resection are pending (3, 4). For limited surgery to be successful, a careful selection of patients who would most benefit from limited surgery is one of the most important steps. However, there are no definite selection criteria for limited resection as of yet; a few studies have suggested possible candidates for limited resection with their study results.

Predominance of ground-glass opacity (GGO) in a lung nodule on computed tomography (CT) has been widely recognized to correlate with less invasive pathological findings of cancer cells replacing the alveolar epithelial cells (5). NSCLC patients with predominantly GGO appearance showed extremely good prognosis following surgical resection (6), suggesting that they are good candidates for limited surgery. In addition, a previous study has also suggested that NSCLC without lymphovascular invasion (LVI) or nodal involvement to be suitable candidates for limited surgical resection. They concluded that consolidation to tumor ratio (C/T ratio) less than 0.25 or 0.5 on a CT scan could accurately predict the absence of LVI or nodal involvement with a very high specificity (96.4%) (7). However, C/T ratio requires a few extra steps of manually measuring the size of the tumor and its solid component and calculating the ratio, which could be time-consuming and increase work burden for the radiologist. If LVI or nodal involvement can be accurately identified with a simpler method, it would prove useful in the selection of candidates for limited resection.

Deep learning has emerged as a powerful tool of representation learning, drastically changing the landscape of feature engineering from hand-crafted manner to a self-taught, machine-driven manner (8). This has been proven to be useful in the field of medical image analysis. Moreover, many studies have successfully demonstrated various applications of deep learning, including nodule detection on chest radiographs (9) and prediction of malignancy in lung nodules (10). A deep learning system can identify features that cannot be assessed by the human eyes. Therefore, we hypothesized that it would be possible to develop a system which would classify and predict pathological features of a nodule on chest CT images to make an accurate selection of possible candidates for limited surgery with a simpler method. Thus, we planned to develop, train, and validate a deep learning algorithm in predicting LVI or pathological nodal involvement using chest CT images without manual measurements.

## Materials and Methods

### Study Population

The clinical and pathological data of NSCLC patients who had undergone curative resection between 2011 and 2015 at two different hospitals (Seoul St. Mary’s Hospital and Incheon St. Mary’s Hospital) of the Catholic University of Korea were reviewed. The inclusion criteria were as follows: (i) pathologically confirmed stage I - III NSCLC; (ii) tumor size of ≤ 3 cm on pathology report; (iii) availability of pathology report; (iv) no preoperative radiation or chemotherapy; (v) availability of chest CT scan (axial images) prior to surgical treatment; and (vi) measurable cancer lesion on preoperative CT images. This study was approved by the institutional review board of Catholic Medical Center (No. UC17SESI0073), and was performed in accordance with the guideline of human research. The requirement for written informed consent was waived by the institutional review board (Catholic Medical Center) because of this study’s character of retrospective analysis.

**Figure 1:**
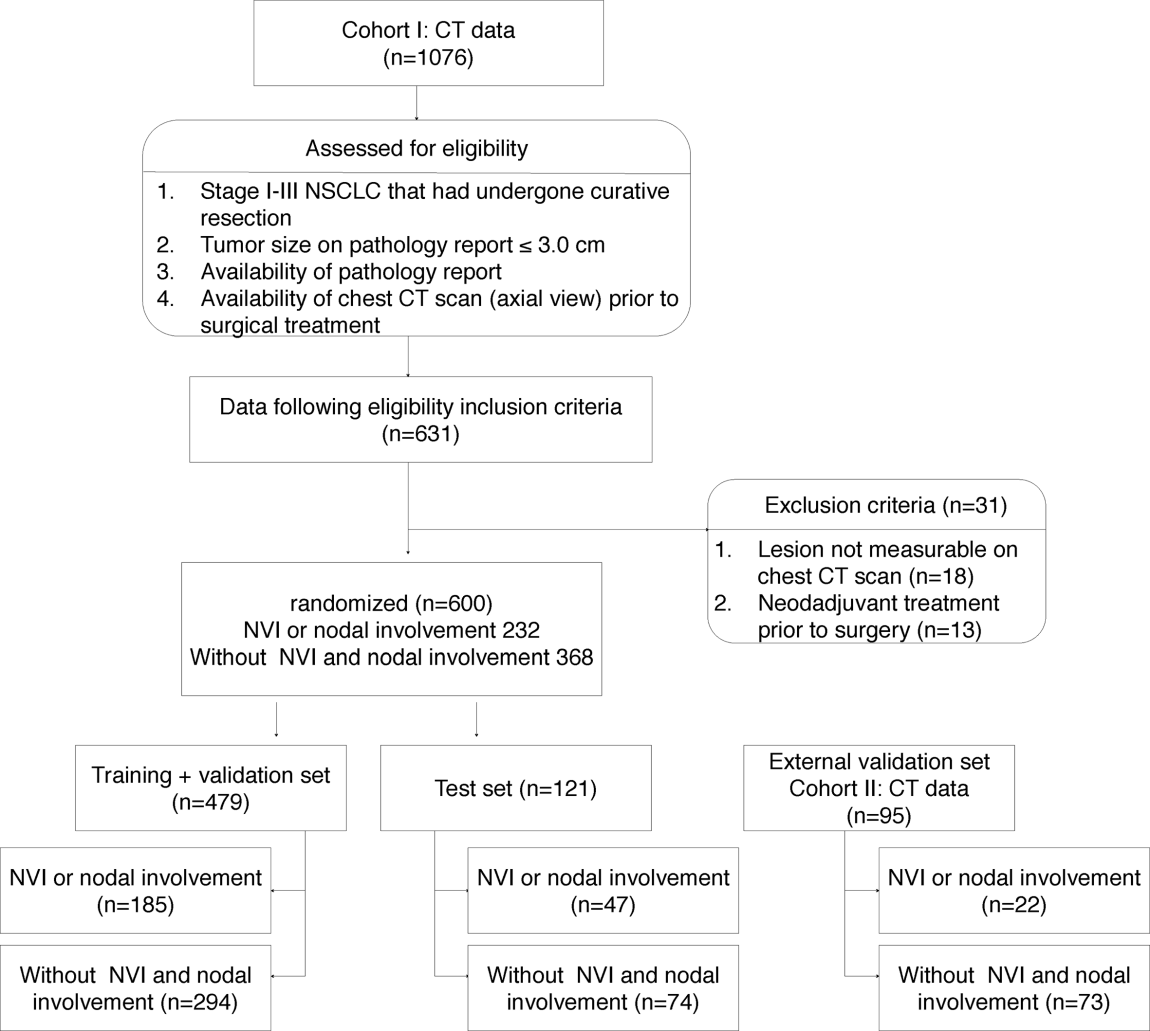
Data criteria and specification.

One thousand seventy-six patients underwent lung cancer surgery between 2011 and 2015, and the final 600 patients who met the inclusion criteria were identified at Seoul St. Mary’s Hospital (cohort I). Patients in cohort I were used for training, validation and test, and 95 patients from Incheon St. Mary’s Hospital (cohort II) were used for external validation (**Figure 1**). Six hundred ninety-five patients (mean age, 63.0 years ± 9.7) were enrolled in this study; the patient cohort consisted of 361 males and 334 females. Two hundred fifty-four (36.5%) had LVI or nodal involvement and 441 (63.5%) no LVI or nodal involvement (**Table 1**).

**Table 1:** Baseline characteristics of the training, validation, and external validation cohorts.

Characteristic		Total (n = 695)	Training and validation cohort (n = 600)	External validation cohort (n = 95)	*p-value*
Age (years)^*^	Mean ± SD	63.0 ± 9.7	63.0 ± 9.8	63.6 ± 9.5	0.551
Sex	Male	361	308 (51.3%)	53 (55.8%)	0.419
	Female	334	292 (48.7%)	42 (44.2%)	
Smoking history	Never	412	349 (58.2%)	63 (66.3%)	0.281
	Current	128	112 (18.7%)	16 (16.8%)	
	Former	155	139 (23.1%)	16 (16.8%)	
Histology	AC	471	395 (65.8%)	76 (80.0%)	0.005
	SqCC	123	108 (18.0%)	15 (15.8%)	
	Others	101	97 (16.2%)	4 (4.2%)	
Tumor size (cm)^†^		2.0 (1.6-2.6)	2.0 (1.5-2.6)	2.1 (1.7-2.6)	0.184
C/T ratio^†^		1.0 (0.5-1.0)	1.0 (0.5-1.0)	1.0 (0.7-1.0)	0.008
LVI or nodal involvement	Yes	254	232 (38.7%)	22 (23.2%)	0.004
	No	441	368 (61.3%)	73 (76.8%)	

^*^Data are mean ± SD.

^†^Measured on CT image and data are median (with interquartile range in parentheses).

C/T ratio, consolidation to tumor ratio; LVI, lymphovascular invasion; AC, adenocarcinoma; SqCC, squamous cell carcinoma.

### Data Preparation and Lesion Labeling

The preoperative CT images at Seoul St. Mary’s Hospital were acquired from Siemens (Somtatom; Erlangen, Germany), with a tube voltage of 120 kVp and tube-current time product of 35-290 mAs, and the images were reconstructed with a slice thickness of 3-5 mm and increment of 3-5 mm. The preoperative CT images at Incheon St. Mary’s Hospital were acquired from Toshiba (Aquilion; Tochigi-ken, Japan), with a tube voltage of 120 kVp and tube-current time product of 30-108 mAs, and the images were reconstructed with a slice thickness of 3-5 mm and increment of 3-5 mm.

Two board-certified radiologists (K.S.B. and B.M.K.), who were blinded to clinical data of all patients, manually drew a rectangular region of interest (ROI) (smallest possible rectangle that could encompass the entire tumor) around the cancer lesion on axial CT images on the PACS workstation (Maroview 5.4; Infinitt, Seoul, Korea) independently. ROI was drawn on the contrast-enhanced images, if available. The chest CT images were extracted in DICOM (Digital Imaging and Communications in Medicine) format with ROI information to develop prediction models.

### Data Splitting and Pretreatment

**Figure 2:**
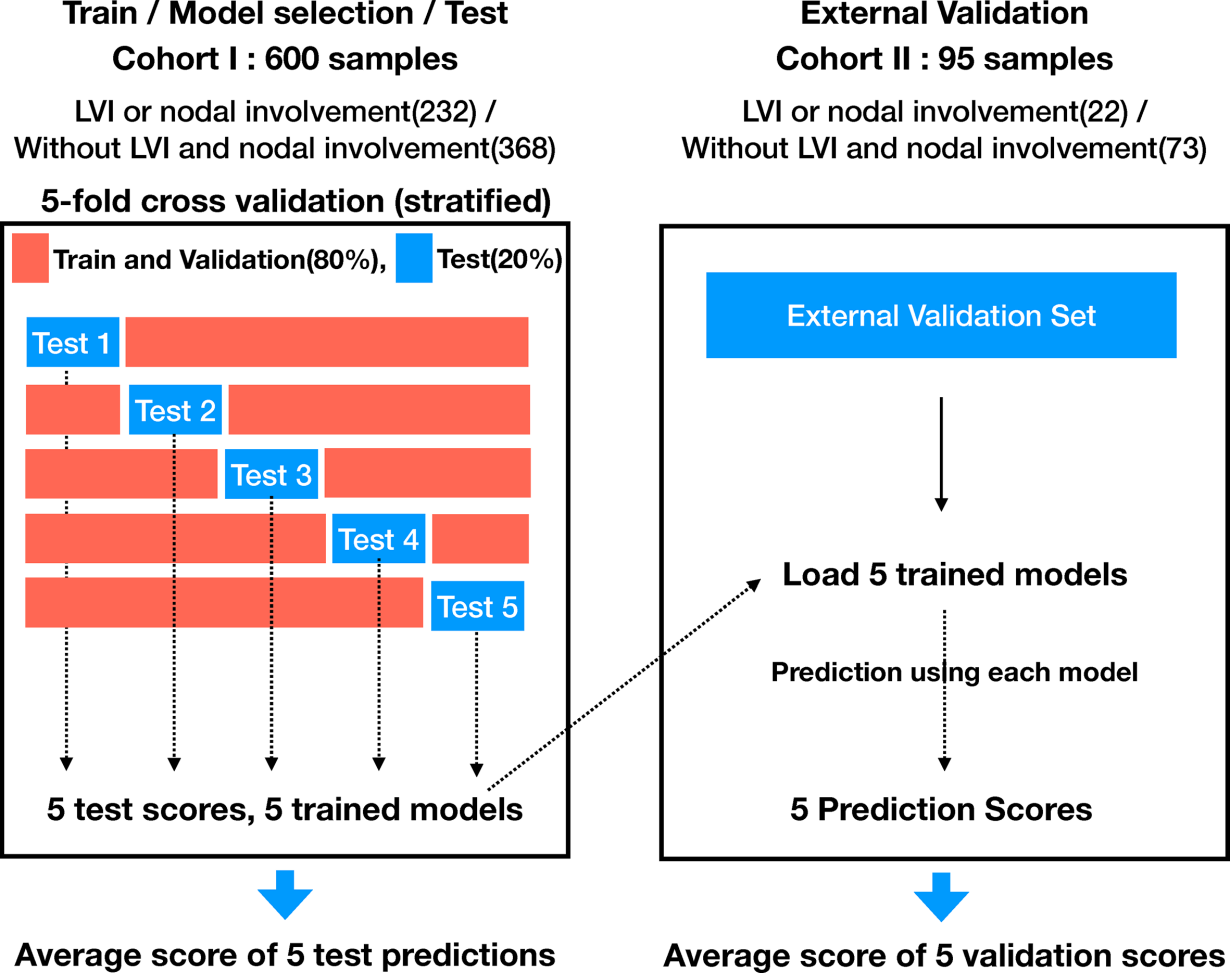
Evaluation pipeline for proposed model.

The cohort I was randomized to maintain the ratio of training (64%), validation (16%), and test (20%). The training and validation sets were used for model learning and optimal model selection, and the test set was used to evaluate the performance of the model. Five-fold stratified cross validation was adopted for training and validation. **Figure 1** indicates a detailed number of the lesions for training, validation, and testing, whereas **Figure 2** shows the overall evaluation pipeline. In the development of the deep learning system, each data sample was defined as (1): A 3D patch of 32mm×32mm×32mm, cropped from the CT scan at the center of a nodule; (2) The pathologically identified label of LVI or nodal involvement; and (3) Manually labeled voxel-wise nodule mask. Online data augmentation (randomly flipping the images on x, y, z axes) was performed for efficient training of the networks.

### Deep Cubical Nodule Transfer Learning (DeepCUBIT) Model

**Figure 3:**
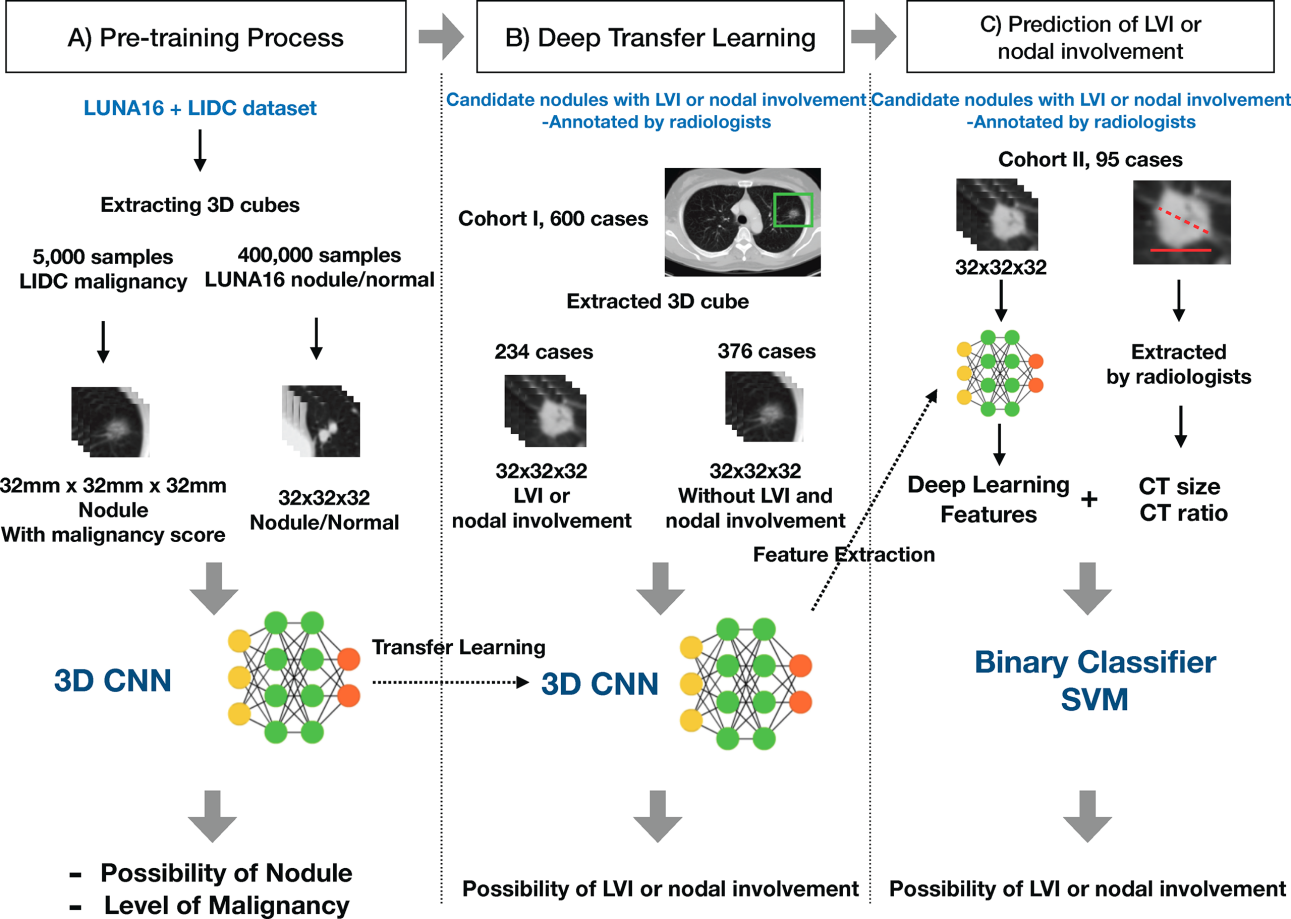
Overall process of the DeepCUBIT algorithm. **(A)** Pre-training Process: Nodule samples and malignancy samples are presented to pre-train. **(B)** Deep Transfer Learning: Predicting the LVI or nodal involvement by fine-tuning the model with weights of pre-trained weights. **(C)** Prediction of LVI or nodal involvement: Feature integration and prediction of LVI or nodal involvement for extra validation cohort.

In order to overcome the limited number of samples of medical data in this study, which we considered insufficient to learn from scratch, we used a transfer learning method. Transfer learning is a machine learning technique for predictive modeling on related tasks that can be reused to accelerate the training and improve the performance of a model. This is done by fine-tuning the weights from a pre-trained network (11). We named the overall process consisting of pre-training, deep transfer learning, and actual prediction of LVI or nodal involvement as DeepCUBIT (Deep CUBical nodule Transfer learning) algorithm (**Figure 3**).

#### Preprocessing

Images were obtained as three-dimensional CT image data by layering two-dimensional slice images. Preprocessing was required to apply the data to the DeepCUBIT model, because the relative size of one voxel is different between samples and the entire CT image cannot be used as an input for the DeepCUBIT model. The CT images were preprocessed in the following steps. In CT scans, each 3D voxel intensity is expressed as Hounsfield Units (HU), which represent a measure of radiodensity. For example, HU value of -1000 represents the air and HU value from -500 to -600 represents the lungs. Inconsistency of cylindrical scanning boundary and image boundary results in an abnormal HU value of - 2000 HU. However, since these are noises and because we thought there is no need to differentiate air and noise, we changed the value of noise to be -1000HU to represent air, instead of original -2000HU. In order to have comparability among samples, we have rescaled the CT images so that one voxel represents size of 1mm×1mm×1mm by linear interpolation, and the voxel values were normalized using min-max normalization for each sample. We extracted 3D nodule cubes in the ROI according to manually annotated center of nodules for the test set, as in the training set. Whole raw image data were used instead of segmenting the lung region because there were nodules on the boundary between lung and mass.

#### Model Architecture

**Figure 4:**
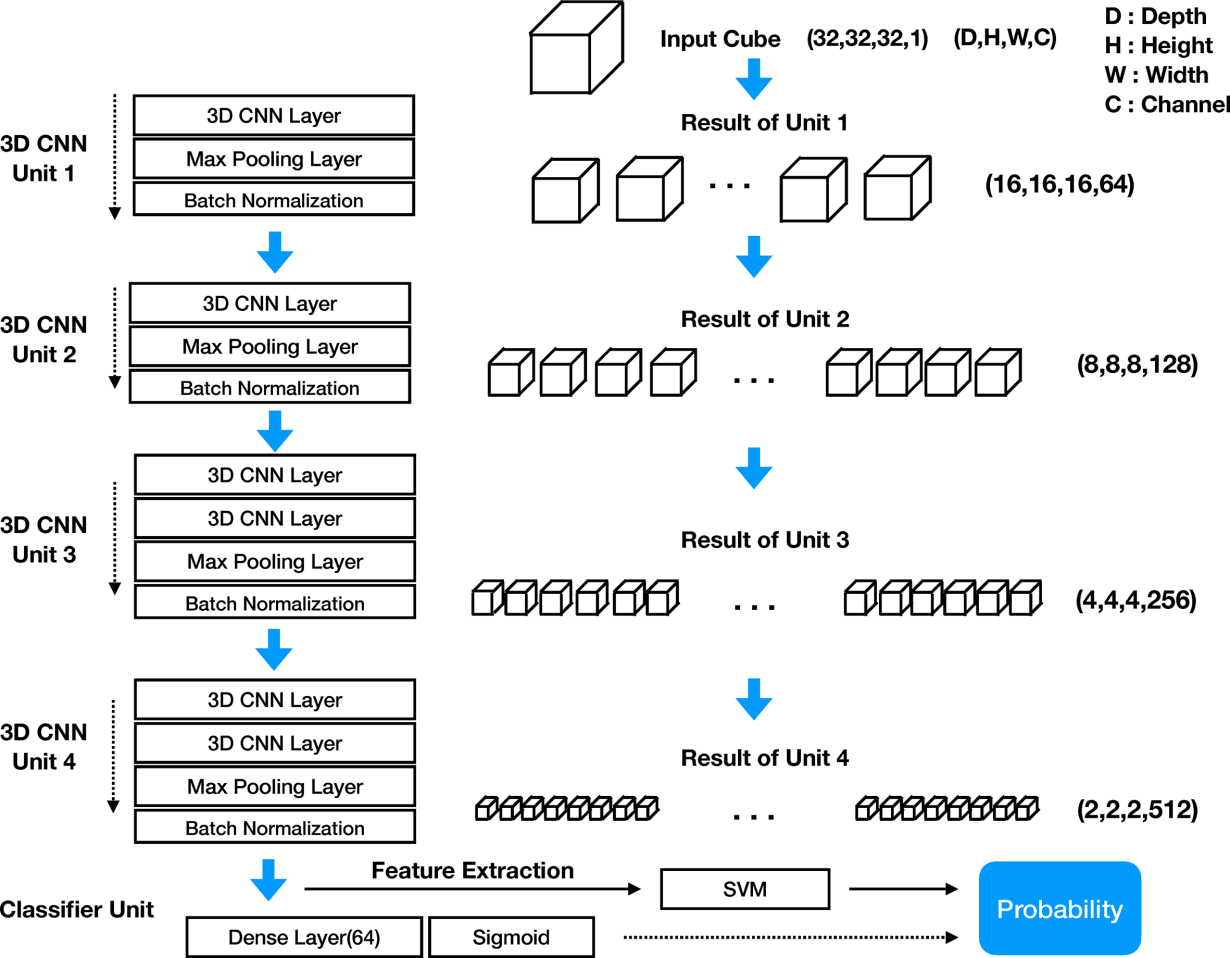
Architecture of DeepCUBIT model.

The structure of the DeepCUBIT consists of four 3D CNN units and one classifier unit. **Figure 4** shows the entire architecture of the DeepCUBIT. In the 3D CNN units, the channels are increased through the convolutional operation of CNN layers. The max-pooling layer is used to reduce resolution (12), and the batch normalization layer (13) is used to speed up the learning time and facilitate the learning process. The kernel size of the CNN layer is all (3, 3, 3), which means 3 pixels depth, 3 pixels height and 3 pixels width. In a 3D CNN layer, adding one kernel increases one channel, and the value computed through convolutional operation using the kernel forms one 3D cube channel. 3D CNN units are stacked to increase the channels and reduce the resolution. Thus, the DeepCUBIT model learns from the detailed features to the high-level abstract features. The classifier unit was made by adding a 64 nodes dense layer and sigmoid layer on the CNN layer. In the transfer learning process, the architecture of all units was unchanged except for the classifier unit. After the training of the model, we changed the classifier unit to support vector machine (SVM) algorithm (14).

#### 3D Convolutional Neural Network

CNN is a type of deep learning model, and some CNN models have the capability of end to end learning (15). In two-dimensional still image data, CNN image filter becomes a 2D kernel acting as a spatial feature extractor. However, in the CT data, we have to reflect the volume information as well as 2D spatial information; therefore, it is necessary to extract the features through the 3D kernels and 3D CNN. The 3D CNN obtains a feature map through a Width × Height × Depth filter called a kernel. The CNN layer creates a new feature map from neighboring pixels centered on the location where the kernel mapping to the previous CNN layer. The size of the feature map itself is reduced by attaching the max pooling layer to the end of the CNN layer. Moreover, stacking more than 2 layers of CNN layer will increase the feature map channel numbers (16). We can get the *n_th_* channel feature maps value of (x, y, z) position of *L_th_* CNN layer from the *L*–1*_th_* CNN layer using the following formula (12).

$$X_{nL}^{xyz}\;=\;f(b_{nL}\;+\;\sum_{m}\;\sum_{p=0}^{Pn-1}\;\sum_{q=0}^{Qn-1}\;\sum_{r=0}^{Rn-1}\;W_{nLm}^{pqr}X_{(L-1)m}^{\left(x+p)(y+q)(z+r\right)})$$

In this formula, f is the activation function of the each node, *b_nL_* is the bias values mapping kernel weights, *P_n_, Q_n_, R_n_* are the size of the 3D kernel consist of width, height, and volume dimension, respectively, and $w_{nLm}^{pqr}$ is the (*p*, *q*, *r*)*_th_* value of the kernel connected to the *m_th_* feature map in the *L*–1*_th_* layer (previous layer). One 3D kernel has a feature map that extracts one feature because one 3D kernel with *P* × *Q* × *R* weights will apply the same weight to all input CT data sliding with fixed stride hops. Therefore, we have to create a large number of kernels to extract various types of features.

#### Deep Transfer Learning

Pre-training domains related to the fine-tuning domain, which predicts LVI or nodal involvement of a nodule in this case, need to be selected for transfer learning to work. Pre-training was on the 3D ROIs. We selected two types of domain for pre-training: a nodule detection domain and a nodule malignancy prediction domain. LUNA16 (LUng Nodule Analysis 2016 challenge, https://luna16.grand-challenge.org/) data set of about 400,000 samples was trained for the first pre-training step to predict the presence of a nodule for the presented 3D cube. LIDC (The Lung Image Database Consortium image collection, https://wiki.cancerimagingarchive.net/display/Public/LIDC-IDRI) data set of about 5,000 samples was trained for the second pre-training step to predict malignancy scores for the presented nodule cubes. The number of samples represents the number of nodule cubes. DeepCUBIT was then fine-tuned using nodule samples of cohort I based on the weights of pre-trained results.

### Predicting Lymphovascular Invasion or Nodal Involvement and Addition of Clinical Data

To assess how well the features are extracted through the deep learning model and how well LVI or nodal involvement of nodule is predicted, we compared the prediction using DeepCUBIT model with prediction using tumor size and C/T ratio. The tumor size and C/T ratio are known indicators for determining the invasiveness of a nodule, both measured on CT and then calculated by the radiologists. We measured the performance of the learned model by using each feature set independently. We also measured the performance of the learned model by combining all the feature sets. We trained the SVM classifier to predict LVI or nodal involvement of a nodule. Radial Basis Function (RBF) kernel was used to train the SVM. The integrated features were used in the training of the SVM classifier after concatenating 64 size vector of DeepCUBIT features, tumor size, and C/T ratio. The process of the final LVI or nodal involvement prediction is presented in a part C) on the **Figure 3**.

Model performance was evaluated by averaging the scores of five stratified hold-out test sets on three different classifiers (SVM, xgboost, random forest). All classifiers were trained using default parameters, and software package versions were as follows: “scikit-learn” python package 0.21.3 for SVM and random forest and “xgboost” python package version 0.90 for xgboost. SVM classifier was finally selected, because it showed the best performance score among the three classifiers.

### Statistical Analysis

Clinicopathologic characteristics are presented as median (range) for continuous variables or numbers (percentage) for categorical variables. Comparisons between the two groups were performed using the Students unpaired t-test or chi-square test. Recurrence-free survival (RFS) was defined as the duration between the date of diagnosis and the date of recurrence date or death from any causes. The performance of the algorithms was evaluated using Harrells concordance-index (C-index), which is a non-parametric statistic that measures concordance between predicted risk and actual survival (17). The predictive performance of all models was compared based on the mean AUC. The evaluation matrix includes Accuracy, Sensitivity, Specificity, PPV (Positive Predictive Value) and NPV (Negative Predictive Value). Kaplan-Meier method and log-rank test were used to determine the differences of estimated survival curves according to the classifier.

## Results

### Performance of Transfer Learning

DeepCUBIT model was pre-trained using a total of 405,000 samples from LUNA16 and LIDC models. As depicted in **Table 2**, DeepCUBIT showed a much better performance than Deep 3D CNN without transfer learning. This result demonstrates that transfer learning is a critical step in training the domains that predict LVI or nodal involvement of nodules.

**Table 2:** Performance comparison for transfer learning in Cohort 1 and 2.

Cohort	P-value	Model	CLF	AUC	Cis (95%)
**1**	**2.447e-09**	**Deep 3D CNN with TL**	NN	**0.682**	** 0.587 - 0.772**
		Deep 3D CNN without TL	NN	0.606	0.503 **-** 0.707
**2**	**7.485e-11**	**Deep 3D CNN with TL**	NN	**0.669**	** 0.553 - 0.78**
		Deep 3D CNN without TL	NN	0490	0.364 **-** 0.625

CNN, Convolutional Neural Network; CLF, Classifier; NN, Neural Network; TL, Transfer Learning; AUC, area under the curve; CIs, Confidence Intervals for AUC score. Variables with DeepCUBIT model are shown in bold type.

### Model Performance

After transfer learning, nodule image features were extracted by the proposed deep network (DeepCUBIT), and clinical features were integrated into the model. The integrated model was performed to predict the performance of the best result in the test sets, and the results of the comparison are reported in **Table 3**, **Supplementary Table 1**, and **Supplementary Figure 1**. Three single SVM classifier models, using 3D CNN, tumor size, and C/T ratio showed AUCs of 0.723, 0.657 and 0.742, respectively. Evaluation scores improved (sensitivity of 75.8%, and specificity of 67.6%, accuracy of 70.8% and AUC of 0.77) after applying SVM to the merged features of DeepCUBIT model, C/T ratio, and tumor size.

**Table 3:** Performance evaluation for test data (Cohort I, average of 5 fold hold-out test set).

Classifier	SVM		Xgboost		Random Forest	
Feature Type	AUC	CIs (95%)	AUC	CIs (95%)	AUC	CIs (95%)
**3D CNN (DeepCUBIT)**	**0.723**	**0.633 - 0.814**	**0.730**	**0.642 - 0.816**	**0.715**	**0.622 - 0.802**
Tumor size	0.657	0.558 - 0.751	0.621	0.522 - 0.720	0.577	0.473 - 0.684
C/T Ratio	0.742	0.663 - 0.817	0.726	0.644 - 0.803	0.631	0.538 - 0.721
Tumor Size + C/T Ratio	0.754	0.669 - 0.834	0.735	0.658 - 0.817	0.686	0.591 - 0.777
3D CNN + Tumor size + C/T Ratio	0.770	0.681 - 0.852	0.752	0.663 - 0.833	0.725	0.635 - 0.813

CNN, Convolutional Neural Network; DeepCUBIT, Deep Cubical Nodule Transfer Learning Algorithm; C/T Ratio, consolidation to tumor ratio; SVM, Support Vector Machine; AUC, area under the curve; CIs, Confidence Intervals for AUC score. Variable with DeepCUBIT model is shown in bold type.

We also did a subgroup analysis in patients with C/T ratio < 1.0 to compare the probability scores of DeepCUBIT and SVM classifier models using C/T ratio in predicting LVI or nodal involvement. The performance of DeepCUBIT model was superior to the C/T ratio model in this subgroup, and the detailed results can be seen in the **Supplementary Table 2**. These findings indicate that deep learning features and clinical features are complementary to each other.

### External Validation

To further analyze the robustness, reproducibility, and reliability of the model, we performed an additional validation using data from cohort II. Similar to the results of cohort I, DeepCUBIT showed the best single model performance (AUC of 0.717) compared to the models using only C/T ratio or tumor size on SVM classifier (**Table 4**, **Supplementary Table 3** and **Supplementary Figure 2**). Applying SVM using all the features, including DeepCUBIT features, tumor size, and C/T ratio, showed the best predictive performance (sensitivity of 31.8%, and specificity of 89.8%, accuracy of 76.4% and AUC of 0.759).

**Table 4:** Performance evaluation for external validation data (Cohort II, external hold-out set).

Classifier	SVM		Xgboost		Random Forest	
Feature Type	AUC	CIs (95%)	AUC	CIs (95%)	AUC	CIs (95%)
**3D CNN (DeepCUBIT)**	**0.717**	**0.601 - 0.819**	**0.685**	**0.566 - 0.797**	**0.660**	**0.528 - 0.779**
Tumor size	0.630	0.502 - 0.749	0.634	0.510 - 0.752	0.606	0.476 - 0.729
C/T Ratio	0.683	0.614 - 0.743	0.682	0.612 - 0.743	0.658	0.591 - 0.733
Tumor size + C/T Ratio	0.716	0.606 - 0.813	0.715	0.613 - 0.812	0.663	0.544 - 0.776
3D CNN + Tumor size + C/T Ratio	0.759	0.646 - 0.855	0.757	0.654 - 0.843	0.716	0.607 - 0.820

CNN, Convolutional Neural Network; DeepCUBIT, Deep Cubical Nodule Transfer Learning Algorithm; C/T Ratio, consolidation to tumor ratio; SVM, Support Vector Machine; AUC, area under the curve; CIs, Confidence Intervals for AUC score. Variable with DeepCUBIT model is shown in bold type.

### Clinical Significance of DeepCUBIT Model

**Figure 5:**
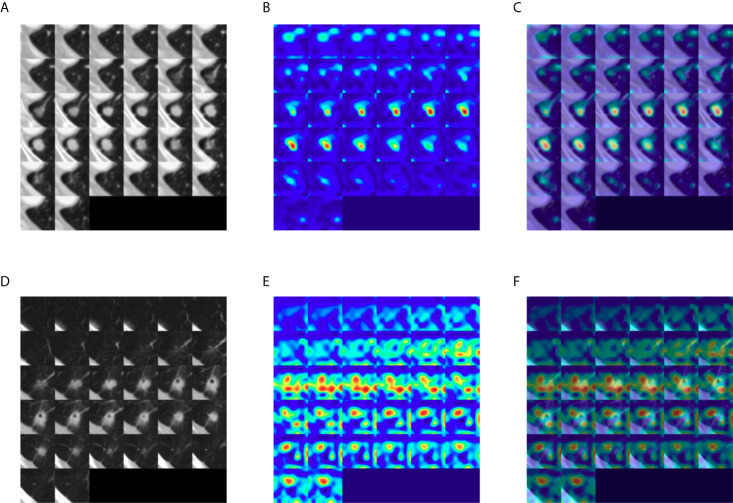
Gradient-weighted class activation heatmaps of nodule cubes. **(A)** Raw intensity, **(B)** gradient heatmap, and **(C)** overaly heatmap of a solid tumor with C/T ratio 1.0 show the area most responsible for the prediction of LVI or nodal involvement to be the solid tumor itself, rather than pleural tag. **(D)** Raw intensity, **(E)** gradient heatmap and **(F)** overlay heatmap of a part-solid tumor with C/T ratio 0.75 show that the area most responsible for the prediction of LVI or nodal involvement to be the interface of the tumor with the adjacent lung parenchyma.

To identify the area in the CT image most responsible for the DeepCUBIT model in predicting LVI or nodal involvement, GradCAM (Gradient-weighted Class Activation Mapping) (18) was adapted to visualize the 3D CNN result, creating heatmaps (**Figure 5**). Grad-cam was applied after the DeepCUBIT network was trained. DeepCUBIT model was made of four 3D CNN blocks. Last output activations and gradients of third 3D CNN layers block was used in the analysis. Heatmaps showed different results for tumors with C/T ratio 1.0 and those with C/T ratio lower than 1.0. In solid tumors with C/T ratio 1.0, the area most responsible for the prediction of LVI or nodal involvement was the tumor itself. However, in part-solid tumors with C/T ratio lower than 1.0, the area most responsible for the prediction of LVI or nodal involvement was the periphery of the tumor, which is the interface between the tumor and the adjacent lung parenchyma.

**Figure 6:**
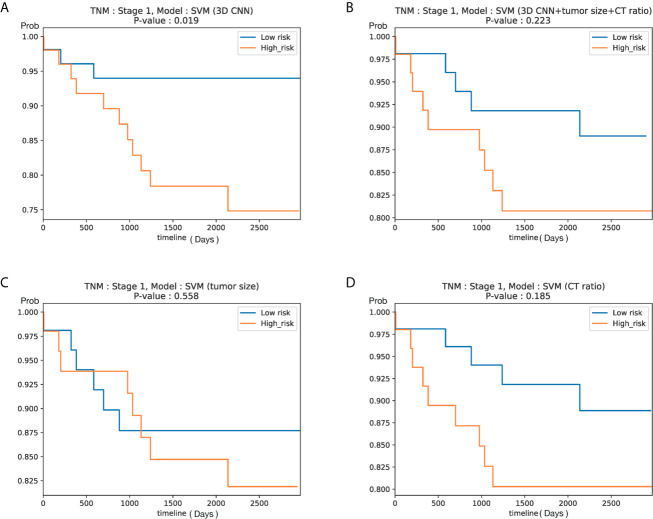
Kaplan–Meier curves according to predicted risk of recurrence for NSCLC patients with stage I in Cohort I (test set only) and Cohort II (105 patients). Curves obtained using **(A)** DeepCUBIT model, **(B)** SVM classifier using DeepCUBIT features with tumor size and C/T ratio, **(C)** SVM classifier using tumor size alone, and **(D)** SVM classifier using C/T ratio alone.

To ascertain the clinical significance of DeepCUBIT model, a novel 3D CNN using the deep cubical transfer learning algorithm, the survival analysis for relapse free survival (RFS) was performed on patients with stage I. These patients were of special interest since postoperative treatment for stage I disease is controversial in external cohort. We assumed that the samples with high invasion probability score will have high risk probability, so we sorted samples according to the invasion probability scores based on the median probability. Despite the small number of patients with stage I disease (105 patients; cohort I, those not used in the training, n = 62; cohort II, n = 43), the RFS of patients with high and low risk scores using DeepCUBIT alone (P = 0.019) and SVM model using DeepCUBIT features with tumor size and C/T ratio (P = 0.223) was significantly different. However, SVM model using tumor size or C/T ratio alone did not demonstrate any significant difference between high and low risk score for the 3-year RFS (**Figure 6**).

## Discussion

Lobectomy is the standard surgical care for patients with resectable NSCLC; recently, pulmonary function-preserving limited surgery has become more prevalent due to the increased number of early and small sized lung cancer, owing to advances in CT technology, more widespread use of CT, and implementation of low-dose CT screening programs worldwide (4). However, previous randomized controlled study had failed to demonstrate the efficacy and validity of limited surgery for clinical early-stage NSCLC (19). Therefore, a careful selection of early stage NSCLC patients for limited resection is of paramount importance to achieve favorable clinical outcomes. One of the factors for favorable clinical outcomes is the absence of LVI or nodal involvement (20). If early-stage NSCLC population without LVI or nodal invasion could be accurately identified before surgery, they could undergo limited resection expecting favorable outcomes. To our knowledge, this the first study incorporating deep learning with preoperative CT images of primary tumor to identify LVI or nodal involvement. We developed a Deep 3D CNN with transfer learning algorithm, the DeepCUBIT, that showed similar performance to the C/T ratio, which is a strong indicator for LVI or nodal involvement of early lung cancer in previous studies (7, 21). Adding C/T ratio and tumor size to the deep learning algorithm further improved the predicting capability of deep learning algorithm. However, even with DeepCUBIT alone, the prediction of LVI or nodal involvement in cT1 stage NSCLC using CT images has become much simpler yet accurate.

The performance of our model, as represented by specificity of 92.6% and sensitivity of 27.6%, is similar to the results of a previous study using C/T ratio to predict LVI or pathological nodal involvement, which resulted in specificity of 96.4% and sensitivity of 30.4% (7). The results cannot be directly compared, because our study population and theirs are slightly different: that study consisted almost entirely of adenocarcinomas (97.1%, to be exact), but our study consisted of 65.8% of adenocarcinomas, and 34.2% were NSCLCs other than adenocarcinomas, and this could have influenced the accuracy.

Lymph node involvement or LVI are known to have higher recurrence rate and mortality risk (20, 22). In previous studies, tumor size and C/T ratio have been identified as well-known risk factors for mediastinal nodal involvement (23). Another study has also shown that the size of the consolidation or solid portion of the primary tumor measured on CT images is one of the independent predictors of lymph node metastasis (24). However, conventional CT images, which rely on lymph node size alone, have a low accuracy in predicting nodal involvement (25). Although the specificity of 18F-FDG PET/CT for detecting lymph node metastasis is high, accuracy of PET-CT is insufficient because of its low sensitivity, especially in the tuberculosis endemic countries (26). Unlike lymph node metastasis, LVI cannot be clinically and preoperatively determined based on CT imaging features. Thus, the fact that LVI or nodal metastasis has been incorporated in the prediction of invasiveness using CT images seems to be an encouraging step. Therefore, our success in using the CT images of the primary tumor to predict LVI or nodal metastasis is in line with the results of previous studies (20, 22).

There have been many conflicting reports dealing with proper indication and efficacy of limited surgery for early stage NSCLC. However, based on the long-term results of the JCOG 0201 trial, limited surgery would lead to satisfactory prognoses in patients with predominantly GGO lung cancers with C/T ratio of 0.5 or less and tumor sizes exceeding 2 cm but 3 cm or less (21, 27). In our study, the performance of the DeepCUBIT model predicting LVI or nodal involvement was similar to that of using C/T ratio, in tumors less than 3cm in size. Of note, this deep learning model can potentially identify patients at high recurrence risk even in stage I patients in a simpler way, which may reflect the biology of primary tumor and provide additional beneficial prognostic information. The benefit from adjuvant chemotherapy is now widely accepted stage in II or III NSCLC (28), but there is no agreement on the use of adjuvant chemotherapy in stage I NSCLC. Previous retrospective study indicated that adjuvant chemotherapy might be beneficial to stage I NSCLC patients with high risk features, such as LVI (29). However, choosing optimal candidates for adjuvant treatment according to conventional single risk factor might be insufficient because it does not consider all clinical or biologic factors and the varying weight of each factor. Thus, we could identify the subgroup harboring a high risk of recurrence that might benefit from adjuvant therapy by applying the novel deep algorithm developed in this study.

In general, to apply the machine learning to CT images, a known feature is extracted from radiologists using the domain knowledge in the CT images or using feature extraction software tools. Then, the machine running is applied to that extracted features. However, in this way, the performance is limited, because new hidden features are difficult to find and learning occurs only within the known existing feature set. To overcome these drawbacks, we used an end-to-end learning method by extracting a feature set from CT images directly using 3D CNN. Moreover, we used transfer learning to improve generality, robustness, and performance. We observed that the addition of transfer learning to 3D CNN improved the performance of 3D CNN, which means that transfer learning process, consisting of pre-training and fine-tuning, is a necessary step in optimization of learning features. Heatmaps generated in order to identify the areas in the CT image most responsible for the DeepCUBIT model in predicting LVI or nodal involvement showed that the DeepCUBIT model seemed to work differently in tumors with C/T ratio 1.0 and those with C/T ratio lower than 1.0. In solid tumors with C/T ratio 1.0, the area most responsible for the prediction of LVI or nodal involvement was the tumor itself. However, in part-solid tumors with C/T ratio lower than 1.0, the area most responsible for the prediction of LVI or nodal involvement was the periphery of the tumor, which is the interface between the tumor and the adjacent lung parenchyma. This suggests that there may be factors not identifiable to the human eye influencing the invasiveness of a part-solid tumor at the interface between the tumor and the adjacent lung parenchyma. Further studies may be needed to expand on this idea. We believe such approach integrating the deep learning models and readily available clinical or radiological data can be used to develop other models in the medical field.

The clinical relevance of the findings in this study has several limitations. First, the number of patients in an independent external validation dataset is relatively small, but both training and validation cohort data, such as radiological findings, standardized surgical treatment, and the detailed records of clinical parameters, were well obtained and of good quality. Second, this is a retrospective study, but the inclusion and exclusion criteria were strictly applied to ensure the inclusion of definite LVI or nodal involvement in the study. Third, there were 18 (2.9%) out of 631 cases with primary cancer lesion “not measurable” on chest CT scans, which were excluded from the study population. All of these lesions were endobronchial lesions in the lobar or intermediate bronchi, and 15 of these tumors could be seen on CT but the exact extent of tumor was not entirely clear because they were blended with distal atelectasis. Three other lesions were diagnosed through transbronchoscopic biopsy but could not be located on CT. However, they only represent a relatively small population (2.9%). Moreover, endobronchial tumors are considered as relative contraindications for performing sublobar resection in NSCLC (30, 31), so we don’t believe excluding these patients would create major limitation for using our approach in the clinical practice for selecting candidates for limited resection. Fourth, the single model of DeepCUBIT did not outperform the boosting model using C/T ratio. This result might be due to the limited number of samples. However, the deep learning model has the advantage that CT images can be directly used to predict LVI or nodal involvement of the tumor without increasing the workload of a radiologist. Fifth, the CT images used in this study are comprised of a heterogeneous mixture of CTs from different vendors, machines, and protocols. Contrast-enhancement images were used if available because we hypothesized enhancement pattern may be useful in determining LVI or nodal involvement. However, because contrast-enhancement images were not available in all patients, the data is heterogeneous, and this could have affected the ability of the deep learning algorithm in predicting LVI or nodal involvement. Nonetheless, we believe such heterogeneity in CT images accurately reflects the real world, and a trained deep learning system with such data may be more fit for the real-world clinical practice.

## Conclusions

The authors have shown that the DeepCUBIT algorithm using transfer learning and 3D CNN based on CT scan images can accurately predict LVI or nodal involvement of primary NSCLC. This deep learning algorithm prediction may be convenient and useful for individualizing treatment modality. In order to predict LVI or nodal involvement of the tumor before surgery, an integrated deep learning approach that combines the multimodal imaging data with clinical data may be more useful.

## Data Availability Statement

The original contributions presented in the study are included in the article/**Supplementary Material**. Further inquiries can be directed to the corresponding authors.

## Ethics Statement

The studies involving human participants were reviewed and approved by the ethical committee at Seoul St. Mary’s Hospital and Incheon St. Mary’s Hospital of the Catholic University of Korea. Written informed consent was waived by the institutional review board because of this study’s character of retrospective analysis.

## Author Contributions

YK and SP generated the concept of the work. BG, SN, SC, JH, JK, SH, WS, HA, and KB performed the data acquisition, analysis, and interpretation. WS, BL, and SP created the new algorithm used in this work. KB drafted the manuscript. HA and YK substantively revised it. All authors contributed to the article and approved the submitted version.

## Funding

This study was supported by a grant from the National R&D Program for Cancer Control, Ministry of Health & Welfare, Republic of Korea (1720100).

## Conflict of Interest

Authors BL, WSS, and SP were employed by company Deargen Inc.

The remaining authors declare that the research was conducted in the absence of any commercial or financial relationships that could be construed as a potential conflict of interest.

## Supplementary Material

Supplementary Figure 1 Comparison of performance of three models (DeepCUBIT, tumor size, and C/T ratio) *via* ROC AUC. AUC scores were calculated using the hold-out test data in cohort I (n = 121 patients, **(A)** and cohort II (n = 95 patients, **(B)**.
