# Supplementary figures and images for: DeepCUBIT: Predicting Lymphovascular Invasion or Pathological Lymph Node Involvement of Clinical T1 Stage Non-Small Cell Lung Cancer on Chest CT Scan Using Deep Cubical Nodule Transfer Learning Algorithm
Source: Front Oncol. 2021 Jul 5;11:661244. doi: 10.3389/fonc.2021.661244 (PMC8287408; doi:10.3389/fonc.2021.661244)

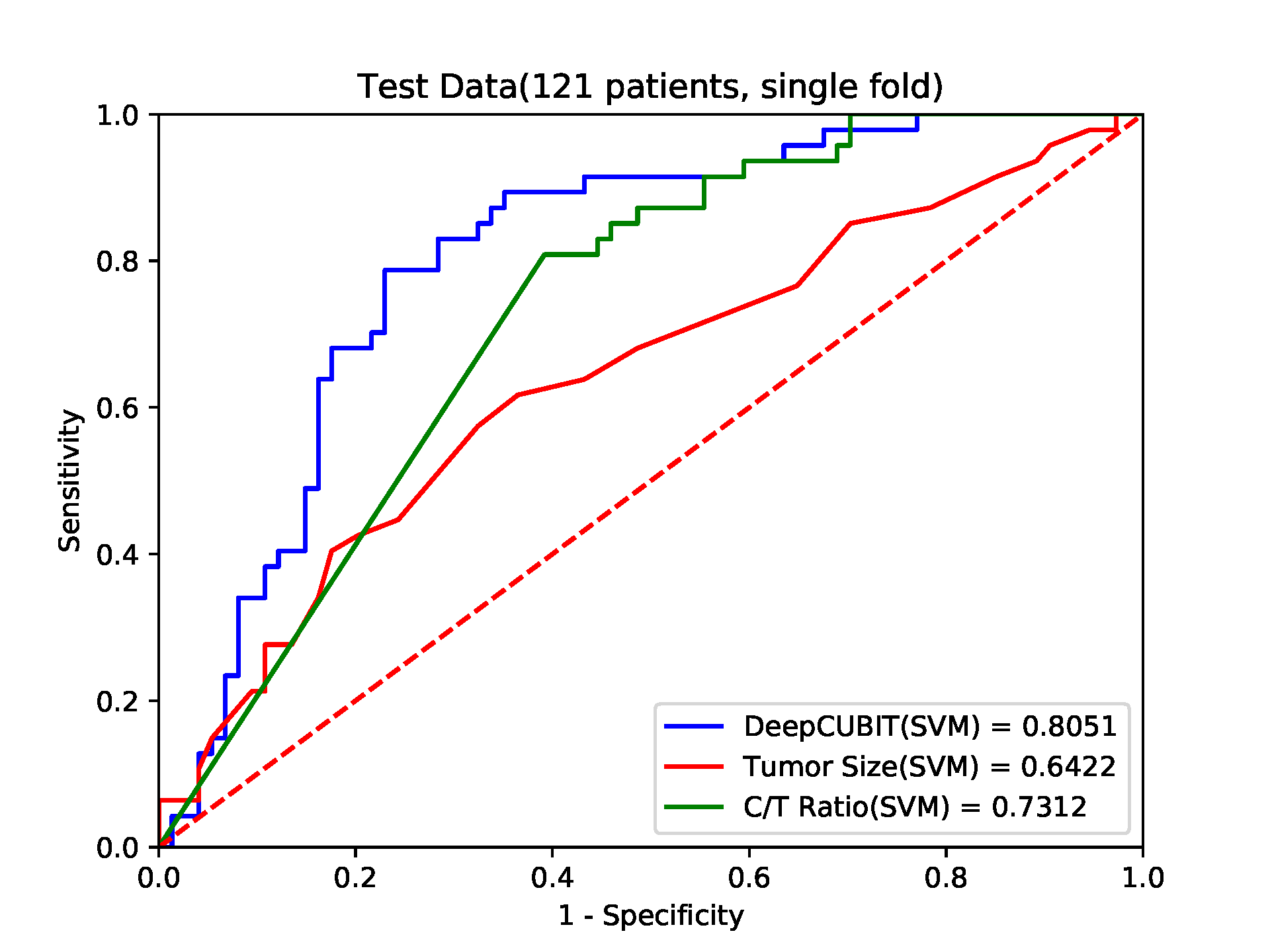

Supplement: Supplementary Figure 1 — Comparison of performance of three models (DeepCUBIT, tumor size, and C/T ratio) via ROC AUC. AUC scores were calculated using the hold-out test data in cohort I (n = 121 patients, (A) and cohort II (n = 95 patients, (B). [file Image_1.tif]

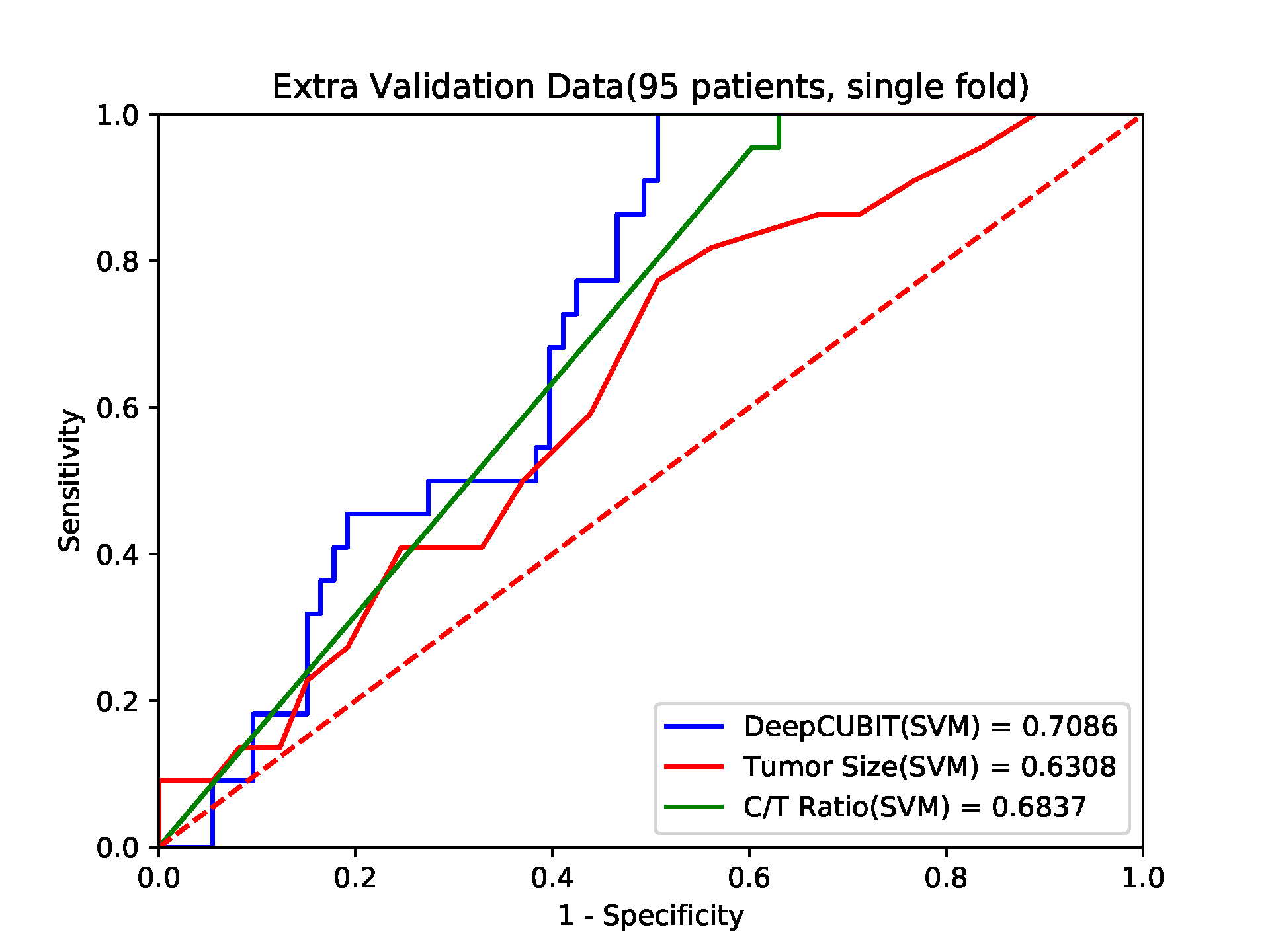

Supplement: Supplementary file 2 [file Image_2.tif]
