# Supplementary material for: DeepCUBIT: Predicting Lymphovascular Invasion or Pathological Lymph Node Involvement of Clinical T1 Stage Non-Small Cell Lung Cancer on Chest CT Scan Using Deep Cubical Nodule Transfer Learning Algorithm
Source: Front Oncol. 2021 Jul 5;11:661244. doi: 10.3389/fonc.2021.661244 (PMC8287408; doi:10.3389/fonc.2021.661244)
Supplement: Supplementary file 3 [file Table_1.docx]

Supplementary Table S1. Evaluation matrix for Cohort I, using variational positive cut off threshold on DeepCUBIT (SVM classifier) model.

| Threshold | Sensitivity (%) | Specificity (%) | PPV (%) | NPV (%) | Accuracy (%) | AUC |
| --- | --- | --- | --- | --- | --- | --- |
| 0.2 | 0.952 | 0.374 | 0.490 | 0.928 | 0.598 | 0.770 |
| 0.4 | 0.758 | 0.676 | 0.601 | 0.816 | 0.708 | 0.770 |
| 0.6 | 0.276 | 0.926 | 0.716 | 0.670 | 0.675 | 0.770 |

DeepCUBIT, Deep Cubical Nodule Transfer Learning Algorithm; PPV, Positive Predictive Value; NPV, negative Predictive Value; AUC, area under the curve; C/T Ratio, consolidation to tumor ratio
