# Supplementary material for: DeepCUBIT: Predicting Lymphovascular Invasion or Pathological Lymph Node Involvement of Clinical T1 Stage Non-Small Cell Lung Cancer on Chest CT Scan Using Deep Cubical Nodule Transfer Learning Algorithm
Source: Front Oncol. 2021 Jul 5;11:661244. doi: 10.3389/fonc.2021.661244 (PMC8287408; doi:10.3389/fonc.2021.661244)
Supplement: Supplementary file 4 [file Table_2.docx]

Supplementary Table S2. Probability comparison on samples with C/T ratio < 1.0 and with LVI or nodal involvement

| Sample | C/T ratio | Probability of predicting LVI or nodal involvement | |
| --- | --- | --- | --- |
|  |  | DeepCUBIT (SVM) | C/T ratio (SVM) |
| 1 | 0.720 | **0.315** | 0.312 |
| 2 | 0.646 | **0.396** | 0.252 |
| 3 | 0.770 | **0.432** | 0.358 |
| 4 | 0.413 | **0.302** | 0.119 |
| 5 | 0.582 | **0.577** | 0.208 |
| 6 | 0.340 | **0.228** | 0.092 |
| 7 | 0.753 | 0.221 | **0.343** |
| 8 | 0.655 | **0.486** | 0.259 |
| 9 | 0.385 | **0.386** | 0.108 |
| Mean | 0.585 | **0.371** | 0.228 |

LVI, Lymphovascular invasion; C/T Ratio, consolidation to tumor ratio; SVM, Support Vector Machine
