# Supplementary material for: DeepCUBIT: Predicting Lymphovascular Invasion or Pathological Lymph Node Involvement of Clinical T1 Stage Non-Small Cell Lung Cancer on Chest CT Scan Using Deep Cubical Nodule Transfer Learning Algorithm
Source: Front Oncol. 2021 Jul 5;11:661244. doi: 10.3389/fonc.2021.661244 (PMC8287408; doi:10.3389/fonc.2021.661244)
Supplement: Supplementary file 5 [file Table_3.docx]

Supplementary Table S3. Evaluation matrix for cohort II, using variational positive cut off threshold on DeepCUBIT (SVM classifier) model.

| Threshold | Sensitivity (%) | Specificity (%) | PPV (%) | NPV (%) | Accuracy (%) | AUC |
| --- | --- | --- | --- | --- | --- | --- |
| 0.4 | 0.918 | 0.446 | 0.333 | 0.949 | 0.555 | 0.759 |
| 0.5 | 0.709 | 0.679 | 0.409 | 0.891 | 0.686 | 0.759 |
| 0.6 | 0.318 | 0.898 | 0.515 | 0.815 | 0.764 | 0.759 |

DeepCUBIT, Deep Cubical Nodule Transfer Learning Algorithm; SVM, Support Vector Machine; PPV, Positive Predictive Value; NPV, negative Predictive Value; AUC, area under the curve; C/T Ratio, consolidation to tumor ratio
